# Hypertension incidence according to branched-chain amino acid intake in Brazilian adults: a six-year follow-up of the CUME study

**DOI:** 10.1007/s00394-026-03903-1

**Published:** 2026-03-11

**Authors:** Fernanda Maria Oliveira da Silva, Adriano Marçal Pimenta, Leidjaira Lopes Juvanhol, Helen Hermana Miranda Hermsdorff, Josefina Bressan

**Affiliations:** 1 Laboratory of Energy Metabolism and Body Composition, Department of Nutrition and Health, Federal University of Viçosa, Viçosa, MG Brazil; 2 Laboratory of Clinical Analysis and Genomics, Department of Nutrition and Health, Federal University of Viçosa, Viçosa, MG Brazil; 3 Department of Nursing, Federal University of Paraná, Curitiba, Brazil; 4 Department of Nutrition and Health, Federal University of Viçosa, Viçosa, Brazil

**Keywords:** Hypertension, Food consumption, Branched-chain amino acid

## Abstract

**Purpose:**

Recent studies show that dietary intake of branched-chain amino acids (BCAA) may be associated with chronic diseases, including hypertension. This study aimed to evaluate the association between BCAA intake and the incidence of hypertension.

**Methods:**

This longitudinal study included 3192 participants (M = 1067, F = 2125; mean age = 34 years) from the Cohort of Universities of Minas Gerais (CUME Study, 2016–2022), Brazil. BCAA intake was assessed using a validated 144-item semi-quantitative food frequency questionnaire. Participants were classified as an incident case of hypertension when they were free of this disease at baseline and had systolic blood pressure ≥ 140 mmHg, diastolic blood pressure ≥ 90 mmHg, or used antihypertensives or received a diagnosis of hypertension by a physician in at least one follow-up. Crude and adjusted Cox regression models were used to evaluate the association between BCAA intake and incidence of hypertension.

**Results:**

After a median follow-up time of 2.21 years, 213 new cases of hypertension were identified. A direct association was shown between the second tertile of total BCAA (HR = 1.76; 95% CI 1.06–2.90) and isoleucine (HR = 2.04; 95% CI 1.14–3.62) consumption and the incidence of hypertension in males. A direct association was observed between the second tertile of valine (HR = 1.74; 95% CI 1.01–3.02) and leucine (HR = 1.94; 95% CI 1.10–3.41) intake and hypertension incidence among adults aged 30–39 years. Conversely, among younger participants (18–29 years), leucine intake in the second tertile was inversely associated with hypertension (HR = 0.36; 95% CI 0.14–0.89). The main foods that contributed to BCAA intake were unprocessed chicken (16.56%), dairy (16.33%), unprocessed beef (14.98%), fish (7.85%), and beans/lentils (6.44%).

**Conclusion:**

Our findings may provide valuable evidence to support dietary interventions for the primary prevention of hypertension.

**Supplementary Information:**

The online version contains supplementary material available at 10.1007/s00394-026-03903-1.

## Introduction

Hypertension is a major cause of premature death worldwide. An estimated 1.28 billion adults aged 30–79 years worldwide have hypertension, two-thirds living in low- and middle-income countries [1]. According to the National Health Survey, the prevalence of hypertension among adults aged 18 and older in Brazil is 39.2% [2].

Hypertension is the most important modifiable risk factor for cardiovascular disease (CVD) and mortality. Adherence to healthy lifestyle factors is associated with a reduced risk of hypertension in the general population and is crucial in preventing CVD [3]. Healthful habits include regular physical activity, healthy eating habits, adequate sleep, and smoking cessation [4]. Nutrition research has shown that dietary protein and amino acid profiles influence the risk for CVD. One of the core components of healthy lifestyles is diet, and in particular, protein and amino acid profiles are closely related to cardiometabolic health [5].

With the evolution of metabolomic analyses, recent studies have demonstrated that dietary intake of amino acids may be associated with chronic conditions, including obesity [6], diabetes mellitus [7], CVD [8], and hypertension [9, 10]. In this context, branched-chain amino acids (BCAAs) have received particular attention due to their potential role in the development of cardiometabolic disorders. Branched-chain amino acids (BCAA), namely valine, leucine, and isoleucine, are essential amino acids that cannot be synthesized by animals and must be obtained through the diet. These amino acids have hydrophobic side chains and participate in protein synthesis and anabolism [11].

Furthermore, branched-chain amino acids (BCAAs) activate key cellular signaling pathways, notably the mechanistic target of rapamycin complex 1 (mTORC1), which plays a central role in metabolic regulation, vascular homeostasis, and endothelial function. Chronic elevation of circulating BCAA levels can lead to sustained mTORC1 activation, which impairs insulin signaling by downregulating insulin receptor substrates (IRS1 and IRS2), increases oxidative stress, and disrupts nitric oxide synthesis, contributing to endothelial dysfunction and dysregulation of blood pressure. Moreover, BCAA catabolism results in the formation of 3-hydroxyisobutyrate (3-HIB), a valine-derived metabolite that enhances transendothelial fatty acid transport, promoting tissue lipotoxicity and further impairing insulin action [12, 13]. These, in turn, are related to the development of CVD, including hypertension.

Few studies assessed the impact of BCAA intake on hypertension [10, 14], especially with a prospective design [9, 15]. Moreover, most available evidence comes from non-Latin American populations, which limits the generalizability of findings to regions with distinct dietary patterns, such as Brazil. In addition, evidence regarding the association between BCAA intake and hypertension remains limited and inconsistent, particularly in prospective studies.

In this context, this study aimed to prospectively evaluate the impact of BCAA intake on the incidence of hypertension in the adult Brazilian population.

## Methods

### Study design

The Cohort of Universities of Minas Gerais (CUME Study) is an observational, open, concurrent, epidemiological study with a defined population group. The project has been conducted in Brazil since 2016, with students attending seven federal public institutions of higher education in Minas Gerais State. The objective is to assess the impact of the Brazilian dietary pattern and nutritional transition on Non-Communicable Diseases [16].

Participants were recruited every two years, resulting in continuous sample growth with each follow-up wave. Previously recruited participants received new questionnaires (Q_2, Q_4, ., Q_*n*), and new participants received the baseline questionnaire (Q_0). Follow-up questionnaires contained questions regarding changes in lifestyle, dietary habits, health conditions, and disease incidence. The study design, dissemination strategies, and participants’ profiles at the first baseline are described in detail in a previous publication [16].

This study was conducted in accordance with the guidelines of the Declaration of Helsinki. All procedures involving human subjects were approved by the Human Research Ethics Committees of all participating institutions, as follows: Federal University of Minas Gerais (CAAE registration number 07223812.3.3001.5153); Federal University of Viçosa (CAAE registration number 4483415.5.1001.5149); Federal University of Ouro Preto (CAAE registration number 44483415.5.2003.5150); Federal University of Lavras (CAAE registration number 44483415.5.2002.5148); Federal University of Juiz de Fora (CAAE registration number 4483415.5.5133); Federal University of Vale do Jequitinhonha and Mucuri (CAAE registration number 44483415.5.2005.5103), and Federal University of Alfenas (CAAE registration number 4.501.344). Written informed consent was obtained from all participants [16].

### Study population

A total of 5723 participants completed the baseline questionnaire (Q_0) in 2016, 2018, 2020, and 2022. Longitudinal data of the accumulated incidence of hypertension from questionnaires Q_0, Q_2, Q_4, and Q_6 (administered in 2016, 2018, 2020, and 2022) were used to evaluate the association with BCAA intake.

**Fig. 1:**
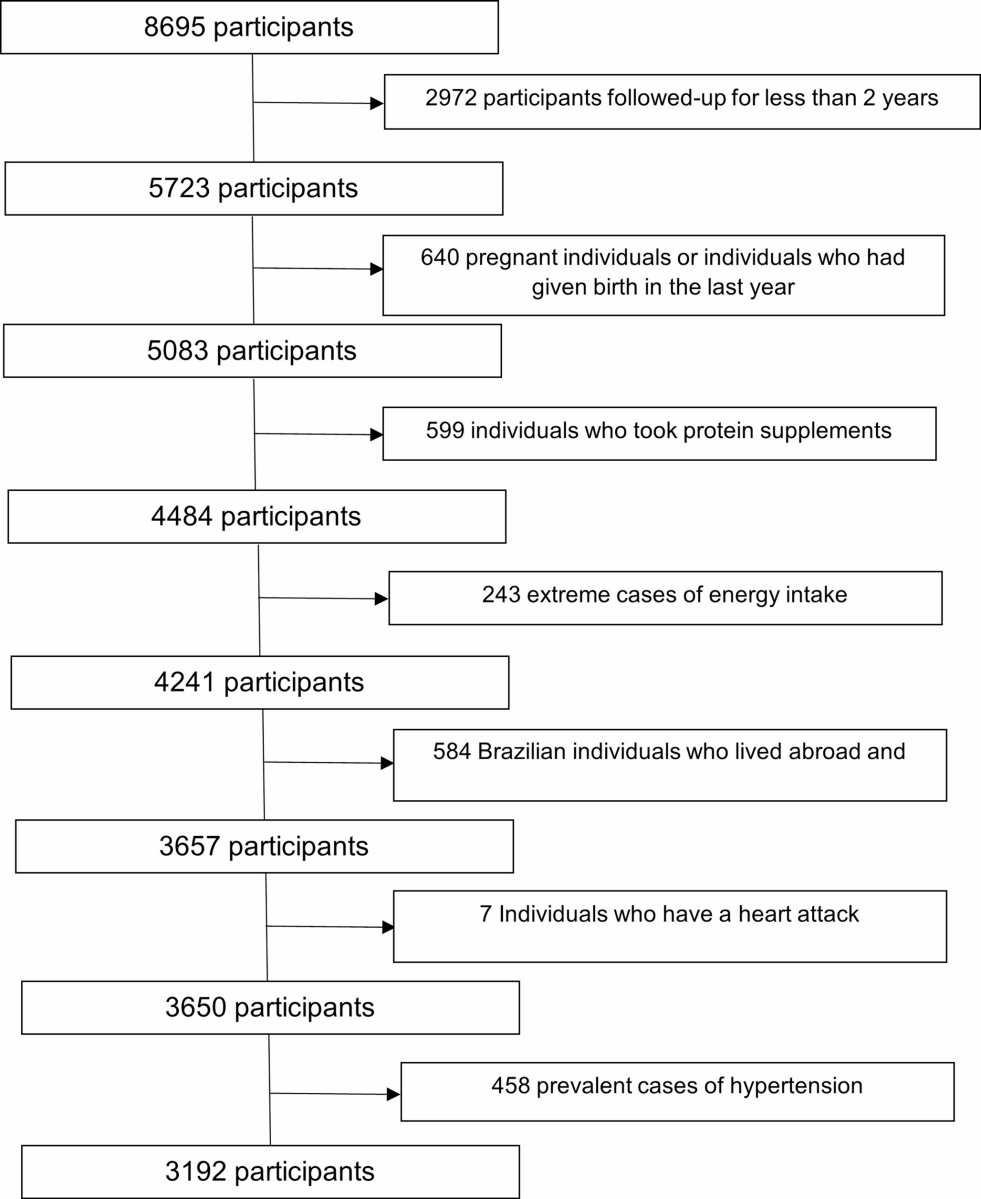
Flowchart of the participant selection process, CUME study (2016–2022)

Exclusion criteria were participants of other nationalities (*n* = 53), Brazilians living abroad (*n* = 531), individuals who had had a heart attack (*n* = 7), extremes of energy intake (< 500 kcal/day or > 6000 kcal/day) (*n* = 243) [17], pregnant individuals and those who had given birth between baseline and follow-up (*n* = 640), individuals who used protein supplements (*n* = 599), and prevalent cases of hypertension (*n* = 458). Thus, the final sample consisted of 3192 participants (Fig. 1).

### Data collection

Data collection was carried out using online questionnaires. The baseline questionnaire (Q_0) consisted of two sections, which were sent separately to participants with a 1-week interval. The first section comprised 83 questions on sociodemographic information, behavioral characteristics, self-reported biochemical tests, medication use, previous diagnoses, and individual and family histories of diseases. The second section consisted of a semi-quantitative food frequency questionnaire (FFQ) comprising 144 items, which was validated for the Brazilian population [18]. A follow-up questionnaire was administered every two years (Q_2, Q_4, and Q_6). This procedure allowed us to examine the incidence of chronic conditions and changes in behavioral habits compared to the baseline (Q_0).

### Outcome variable

In this study, the dependent variable was the incidence of hypertension, which was defined as the proportion of disease-free participants at baseline who were classified as hypertensive at follow-up. Hypertension was determined based on the presence of at least one of the following criteria [19]: systolic blood pressure ≥ 140 mmHg or diastolic blood pressure ≥ 90 mmHg, use of antihypertensives, or a diagnosis of hypertension by a physician in at least one of the follow-ups. These self-reported measures were previously validated in a subsample of 172 participants from the CUME project, demonstrating moderate to substantial agreement with directly measured values. The validation study reported an intraclass correlation coefficient (ICC) of 0.67 for systolic blood pressure (SBP), 0.49 for diastolic blood pressure (DBP), and a kappa statistic of 0.56 for the diagnosis of hypertension [20].

### Exposure variable

The exposure variable was BCAA intake. For data collection, food items were grouped as follows: dairy products; meat and fish, including sausages and eggs; cereals and legumes; fats and oils; fruits; vegetables; beverages (beer and distilled alcoholic beverages, soft drinks, and natural and industrial juices); and other foods (snacks, sweet desserts, salty snacks, sweeteners, sugars, and salt). The frequency of consumption of a given food within the previous year was classified as daily, monthly, weekly, or annual. Participants were also asked to indicate portion sizes. Images of food items and utensils were provided to assist participants in selecting food types and portion sizes, which were expressed in both household measurements and traditional portions [18].

Consumption frequencies were transformed into daily intakes per food item. The daily food intake (grams or milliliters) was calculated by multiplying the portion size by the consumption frequency. Information obtained from the FFQ was used to estimate energy intake, macronutrients, (carbohydrates, proteins, and lipids), leucine, isoleucine, valine, and total BCAA. Dietary variables were energy-adjusted using the residual method [21].

Estimation of food consumption and macronutrient and micronutrient contents was carried out using Dietpro^®^ software version 5i. The software provides the Brazilian food composition Table [22] and the USDA food chemical composition table. Both tables list the amount of amino acids per serving (100 g), which was used as a basis for calculating the portion size of food items. The Brazilian food composition Table [22] was used to compare the nutritional composition of typical Brazilian dishes and preparations. When items were not available in the Brazilian table, the nutritional composition of the preparations was estimated based on the USDA table.

The relative contribution of each food to the daily BCAA intake was calculated as the ratio of the individual nutrient content of each food to the total nutrient content of all foods multiplied by 100 [23]. BCAA intake was expressed in grams and classified into tertiles.

### Covariates

#### Sociodemographic variables

The following sociodemographic variables were determined using the baseline questionnaire: age group (18–29, 30–39, 40–49, 50–59, and ≥ 60 years), sex (female, male), marital status (married/common-law, divorced/separated, single, widowed/other), ethnic group/skin color (White, Brown, Black, Yellow, Indigenous), level of education (doctoral/postdoctoral degree, master’s degree, specialization, undergraduate degree), per capita income (household income divided by the number of individuals in the household), and employment status (retired/homemaker, unemployed, student, full-time, part-time).

#### Behavioral variables

Binge drinking was defined as consuming ≥ 4 doses of alcoholic beverages for women or ≥ 5 doses for men on a single occasion in the last 30 days [24]. Physical activity level was determined by the mean number of days and the mean duration (in minutes) of physical activity practice per week. Intensity was determined using a subjective scale (ranging from 0 to 5 to 10). A list of 23 types of physical activities and sports, with duration expressed in minutes or hours, was provided [25]. Individuals who practiced ≥ 150 min/week of moderate-intensity activity or ≥ 75 min/week of vigorous-intensity activity were considered active. Physical inactivity was defined as the absence of physical activity [26].

#### Disease variable

In the 2022 wave of data collection, the following question was added to assess the diagnosis of COVID-19: “Since the last questionnaire answered in 2020, have you been diagnosed with COVID-19 by a doctor?”

#### Anthropometric variable

Self-reported weight (kg) and height (m) data were used to calculate BMI, which were previously validated by Miranda et al. [20]. Participants with BMI < 25 kg/m^2^ were considered normal-weight and those with BMI ≥ 25 kg/m^2^ were considered overweight [20].

### Statistical analysis

Participant characteristics were expressed as absolute and relative frequencies for categorical variables and median and interquartile range for quantitative variables. Statistical differences were assessed using Pearson’s chi-squared test (categorical variables) and Mann–Whitney or Kruskal–Wallis tests (quantitative variables).

Crude Cox regression models and models adjusted for potential confounders were estimated to assess the association between BCAA intake and the incidence of hypertension. We also conducted additional analyses stratified by sex, age groups, and physical activity levels to explore potential effect modification across these subgroups. Follow-up time was calculated in person-years for each participant, as follows: the difference between the completion date of the follow-up questionnaire in which hypertension incidence was identified and the completion date of the baseline questionnaire; or the difference between the completion date of the last follow-up questionnaire and the completion date of the baseline questionnaire when the outcome was not identified.

Adjustment variables were defined using a directed acyclic graph (Online Resource 1). The first model was adjusted for gender, age, skin color, per capita income, physical activity, excessive alcohol consumption, smoking, saturated fat, carbohydrate intake, salt intake, and BMI. The second model was additionally adjusted for COVID-19.

The association measure used was the hazard ratio (HR), along with its respective 95% confidence intervals (95% CI). Data analyses were conducted using Stata statistical software version 13.1 (https://www.stata.com). The significance level adopted in all analyses was 5%.

## Results

Of the total of 3192 participants, 213 had incident hypertension. The incidence was 15.7/1000 person-years in females, 28/1000 person-years in males, and 19.8/1000 person-years total. The median follow-up time was 2.21 years (IQR: 1.98–4.22 years).

Participants with incident hypertension (Table 1) were more likely to be women (*p* < 0.001), aged ≥ 40 years (*p* < 0.001), with a specialization degree (*p* < 0.001), working full-time or partially (*p* < 0.001), non-smokers (*p* < 0.01), and with higher median energy consumption (*p* < 0.05).

Participants in the highest tertile of BCAA intake (Table 2) were more likely to be physically active, have higher medians of energy, protein, and saturated fat consumption, and have lower medians of carbohydrate consumption (*p* < 0.05). The main foods that contributed to BCAA intake were unprocessed chicken (16.56%), dairy (16.33%), unprocessed beef (14.98%), fish (7.85%), and beans/lentils (6.44%) (Table 3).

**Table 1:** Sociodemographic, lifestyle, and dietary intake characteristics (energy-adjusted) of study participants according to hypertension incidence (CUME study, *n* = 3192, 2016–2022)

Variable	Hypertension incidence			*P*-value ^a,b^
	No	Yes	Total	
	*n* = 2979	*n* = 213	*n* = 3192	
Sex				
Male	967 (32.46)	100 (46.95)	1067 (33.43)	** < 0.001**
Female	2012 (67.54)	113 (53.05)	2125 (66.57)	
Age group (years)				
18–29	903 (30.31)	37 (17.37)	940 (29.45)	** < 0.001**
30–39	1244 (41.76)	78 (36.62)	1322 (41.42)	
40–49	542 (18.19)	61 (28.64)	603 (18.89)	
50–59	236 (7.92)	25 (11.74)	261 (8.18)	
≥ 60	54 (1.81)	12 (5.63)	66 (2.07)	
Skin color				
White	1937 (65.02)	135 (63.38)	2072 (64.91)	0.436
Brown/Black	1003 (33.67)	73 (34.27)	1076 (33.71)	
Yellow/Indigenous	39 (1.31)	5 (2.35)	44 (1.38)	
Level of education				
Undergraduate degree	855 (28.70)	42 (19.72)	897 (28.10)	**0.001**
Specialization	621 (20.85)	65 (30.52)	686 (21.49)	
Master's degree	918 (30.82)	57 (26.76)	975 (30.55)	
Doctoral/postdoctoral degree	585 (19.64)	49 (23.00)	634 (19.86)	
Employment status				
Full-time/partial-time	2174 (72.98)	165 (77.46)	2339 (73.28)	** < 0.001**
Student	539 (18.09)	24 (11.27)	563 (17.64)	
Retired/homemaker	57 (1.91)	13 (6.10)	70 (2.19)	
Unemployed	209 (7.02)	11 (5.16)	220 (6.89)	
Per capita income				
< 5 minimum wages	2155 (72.34)	146 (68.54)	2301 (72.09)	0.271
5–9 minimum wages	631 (21.18)	55 (25.82)	686 (21.49)	
≥ 10 minimum wages	193 (6.48)	12 (5.63)	205 (6.42)	
Smoking				
No	2397 (80.46)	153 (71.83)	2550 (79.89)	**0.010**
Ex-smoker	322 (10.81)	33 (15.49)	355 (11.12)	
Smoker	260 (8.73)	27 (12.68)	287 (8.99)	
Binge drinking				
No	1844 (61.9)	123 (57.75)	1967 (61.62)	0.229
Yes	1135 (38.1)	90 (42.25)	1225 (38.38)	
Physical activity				
Inactive	741 (24.87)	56 (26.29)	797 (24.97)	0.893
Insufficiently active	608 (20.41)	42 (19.72)	650 (20.36)	
Active	1630 (54.72)	115 (53.99)	1745 (54.67)	
BMI (kg/m^2^**)**				** < 0.001**
< 25	1812 (60.83)	81 (38.03)	1893 (59.30)	
≥ 25	1167 (39.17)	132 (61.97)	1299 (40.70)	
COVID-19				
No	488 (65.24)	57 (65.52)	545 (65.27)	0.959
Yes	260 (34.76)	30 (34.48)	290 (34.73)	
Energy and nutriente intake				
Energy intake (kcal/day)	2185.62 (1685.59–2805.93)	2339.79 (1810.02–2991.74)	2199.01 (1693.79–2826.67)	**0.015**
Carbohydrate (g/day)	247.24 (211.49–280.98)	243.24 (212.48–282.61)	247.11 (211.52–281.24)	0.881
Protein (g/day)	240.95 (204.78–282.30)	241.92 (208.68–274.53)	241.02 (205.23–281.81)	0.676
Saturated fat (g/day)	31.15 (25.91–36.64)	31.28 (25.40–36.12)	31.20 (25.89–36.59)	0.788
Salt (g/day)	2.70 (1.44–3.37)	2.74 (1.59–6.94)	2.70 (1.44–3.39)	0.692
BCAAs (g/day)^c^	14.82 (12.30–17.58)	14.49 (12.20–17.25)	14.80 (12.30–17.55)	0.362
Animal-derived BCAA (g/day)	9.66 (6.68–14.10)	9.99 (7.04–15.21)	9.68 (6.70–14.13)	0.168
Plant-derived BCAA (g/day)	4.38 (3.17–6.10)	4.46 (3.44–6.49)	4.38 (3.19–6.13)	0.147
Animal-to-plant BCAA ratio	2.18 (1.43–3.27)	2.22 (1.53–3.31)	2.18 (1.44–3.28)	0.728
Valine (g/day)	4.23 (3.51–5.05)	4.12 (3.53–4.96)	4.22 (3.51–5.04)	0.333
Isoleucine (g/day)	3.73 (2.74–5.07)	3.91 (2.93–5.37)	3.74 (2.75–5.10)	0.134
Leucine (g/day)	6.70 (5.57–7.92)	6.55 (5.64–7.75)	6.69 (5.57–7.95)	0.349

Values in bold indicate statistically significant results (*p* < 0.05)

^a^Results are presented as absolute and relative frequencies or median and interquartile range

^b^Pearson's chi-squared test (categorical variables) or Mann–Whitney test (quantitative variables)

^c^BCAA values correspond to the sum of valine, isoleucine, and leucine intake

BMI, body mass index; BCAA, branched-chain amino acids

**Table 2:** Sociodemographic, lifestyle, and dietary intake characteristics (energy-adjusted) of study participants according to branched-chain amino acid intake (CUME study, *n* = 3192, 2016–2022)

	BCAA intake tertiles			*P* value ^a, b^
	T1 (< 13.24 g)	T2 (13.24–16.42 g)	T3 (> 16.42 g)	
Participantes, N	*n* = 1064	*n* = 1064	*n* = 1064	
Sex				
Female	733 (68.89)	684 (64.29)	708 (66.54)	0.079
Male	331 (31.11)	380 (35.71)	356 (33.46)	
Age group (years)				
18–29	299 (28.10)	307 (28.85)	334 (31.39)	0.080
30–39	417 (39.19)	467 (43.89)	438 (41.17)	
40–49	220 (20.68)	197 (18.52)	186 (17.48)	
50–59	104 (9.77)	74 (6.95)	83 (7.80)	
≥ 60	24 (2.26)	19 (1.79)	23 (2.16)	
Skin color				
White	667 (62.69)	695 (65.32)	710 (66.73)	0.370
Brown/Black	383 (36.00)	353 (33.18)	340 (31.95)	
Yellow/Indigenous	14 (1.32)	16 (1.50)	14 (1.32)	
Level of education				
Undergraduate degree	311 (29.23)	308 (28.95)	278 (26.13)	0.171
Specialization	248 (23.31)	210 (19.74)	228 (21.43)	
Master’s degree	308 (28.95)	323 (30.36)	344 (32.33)	
Doctoral/postdoctoral degree	197 (18.52)	223 (20.96)	214 (20.11)	
Employment status				
Full-time/partial-time	776 (72.93)	784 (73.68)	779 (73.21)	0.995
Student	189 (17.76)	186 (17.48)	188 (17.67)	
Retired/homemaker	25 (2.35)	24 (2.26)	21 (1.97)	
Unemployed	74 (6.95)	70 (6.58)	76 (7.14)	
Per capita income				
< 5 minimum wages	783 (73.59)	779 (73.21)	739 (69.45)	0.173
5–9 minimum wages	219 (20.58)	214 (20.11)	253 (23.78)	
≥ 10 minimum wages	62 (5.83)	71 (6.67)	72 (6.77)	
Smoking				
No	830 (78.01)	857 (80.55)	863 (81.11)	0.089
Ex-smoker	127 (11.94)	105 (9.87)	123 (11.56)	
Smoker	107 (10.06)	102 (9.59)	78 (7.33)	
Binge drinking				
No	664 (62.41)	644 (60.53)	659 (61.94)	0.650
Yes	400 (37.59)	420 (39.47)	405 (38.06)	
Physical Activity				
Inactive	305 (28.67)	270 (25.38)	222 (20.86)	**< 0.001**
Insufficiently active	219 (20.58)	225 (21.15)	206 (19.36)	
Active	540 (50.75)	569 (53.48)	636 (59.77)	
BMI (kg/m²)				0.310
< 25	650 (61.09)	627 (58.93)	616 (57.89)	
≥ 25	414 (38.91)	437 (41.07)	448 (42.11)	
COVID-19				
No	194 (64.24)	175 (64.34)	176 (77.43)	0.675
Yes	108 (35.76)	97 (35.66)	85 (32.57)	
Energy and nutriente intake				
Energy intake (kcal/day)	2063,87 (1485.97–3041.00)	2170.66 (1718.30-2739.73)	2288.03 (1843.87-2814.28)	**< 0.001**
Carbohydrate (g/day)	264.12 (225.21-303.05)	253.00 (222.82-281.21)	225.99 (192.78–260.00)	**< 0.001**
Protein (g/day)	192.18 (169.54-210.61)	242.52 (227.99-258.43)	297.83 (271.99-334.57)	**< 0.001**
Saturated fat (g/day)	28.27 (23.05–33.77)	32.08 (27.63–37.37)	32.84 (27.76–37.90)	**< 0.001**
Salt (g/day)	2.86 (1.79–6.41)	2.67 (1.39–3.21)	2.61 (1.22–3.23)	**< 0.001**

Values in bold indicate statistically significant results (*p* < 0.05)

^a^Results are presented as absolute and relative frequencies or median and interquartile range

^b^Pearson’s chi-squared test (categorical variables) or Kruskal–Wallis test (quantitative variables)

BMI, body mass index

BCAA, branched-chain amino acids

**Table 3:** Contribution (%) of food items to branched-chain amino acid intake among participants (CUME study, *n* = 3192, 2016–2022)

BCAA total			
Food	(%)	Food	(%)
Unprocessed meat		Cream cheese	0.96
Chicken (with/without skin)	16.56	Ricotta	0.40
Beef	14.98	Semi-skimmed milk	1.01
Other fish	5.15	Dairy products (Total)	16.33
Sardine/tuna/salmon/cod	2.70	Eggs	3.51
Fish (Total)	7.85	Others	
Pork	1.77	Beans/lentils	6.44
Lamb	0.14	Peanut/walnut/other nuts	2.93
Offal	0.03	White rice	2.61
Processed meat		Soy milk	1.83
Turkey ham	0.69	Pizza	1.91
Sausage	1.27	Whole bread	1.57
Mortadella	0.78	Cheese bread	1.41
Hot dog sausage	0.34	Lasagna	1.03
Bacon	0.27	Pasta	0,89
Smoked meat	0.17	Sliced bread	0.75
Dairy products		Whole rice	0.56
Cheese	8.25	Sweet bread	0.46
Whole milk	3.46	Soy protein	0.10
Skimmed milk	2.25	Oat/granola	0.09
Total			87.27%

The results of the regression analysis investigating the association between BCAA and hypertension incidence are presented in Table 4 and Online Resource 2. In the general analysis, no independent association was observed between the tertiles of total BCAA intake, valine, leucine, or isoleucine intake and the incidence of hypertension in the adjusted models. In the analysis stratified by sex, there was a direct association between the second tertile of total BCAA and isoleucine consumption and incidence of hypertension for males (Table 5). In the analysis stratified by age group, there was a direct association between the second tertile of valine and leucine intake and the incidence of hypertension among adults aged 30–39 years. In contrast, among younger participants (18–29 years), there was an inverse association between the second tertile of leucine intake and the incidence of hypertension (Online Resource 4). No significant associations were observed in the analysis stratified by physical activity level (data not shown).

**Table 4:** Association between branched-chain amino acid intake tertiles and hypertension incidence (CUME study, *n* = 3192, 2016–2022)

Variables	BCAA intake tertiles			Trend *p*-value
	T1 *n* = 1064	T2 *n* = 1064	T3 *n* = 1064	
Total BCAA (g/day)	< 13.24	13.24–16.41	> 16.41	
HR (95% CI)	1.00	1.20 (0.86–1.67)	0.99 (0.69–1.42)	0.998
Animal-derived BCAA (g/day)	**< 9.30**	**9.30–12.51**	**> 12.51**	
HR (95% CI)	1.00	1.08 (0.73–1.53)	1.04 (0.70–1.54)	0.816
Plant-derived BCAA (g/day)	**< 3.40**	**3.40–10.91**	**> 10.91**	
HR (95% CI)	1.00	0.85 (0.61–1.19)	0.88 (0.62–1.24)	0.429
Valine (g/day)	**< 3.76**	**3.76–4.72**	**> 4.72**	
HR (95% CI)	1.00	1.12 (0.80–1.55)	0.96 (0.67–1.37)	0.876
Leucine (g/day)	**< 5.98**	**5.98–7.46**	**> 7.46**	
HR (95% CI)	1.00	1.12 (0.80–1.57)	1.06 (0.74–1.52)	0.720
Isoleucine (g/day)	**< 3.08**	**3.08–4.57**	**> 4.57**	
HR (95% CI)	1.00	1.06 (0.75–1.50)	1.01 (0.71–1.42)	0.955

Values in bold indicate g/dia

The regression model was adjusted for age, skin color, smoking, per capita income, physical activity, excessive alcohol consumption, saturated fat intake, carbohydrate intake and salt intake

HR, Hazard Ratio

95% CI, 95% Confidence Interval

BCAA, branched-chain amino acids

**Table 5:** Association between branched-chain amino acid (BCAA) intake tertiles and hypertension incidence, stratified by sex (CUME study, *n* = 3192, 2016–2022)

BCAA (g/day)	Men (*n* = 1067)		BCAA (g/day)	Women (*n* = 2125)	
	HR (95% CI)	*p*-Value		HR (95% CI)	*p*-Value
T1:331 (< 13.41)	1.00		T1: 733 (< 13.12 g)	1.00	
T2: 380 (13.41–16.42)	1.76 (1.06–2.90)	**0.026**	T2: 684 (13.12–16.41)	0.84 (0.53–1.33)	0.473
T3: 356 (> 16.42)	1.33 (0.73–2.29)	0.296	T3: 708 (> 16.41)	0.76 (0.48–1.21)	0.260
Valine (g/day)			Valine (g/day)		
T1: 338 (< 3.82)	1.00		T1:726 (< 3.74)	1,00	
T2: 381 (3.82–4.70)	1.50 (0.91–2.44)	0.105	T2: 683 (3.74–4.73)	0.83 (0.52–1.32)	0.442
T3: 348 (> 4.70)	1.20 (0.70–2.05)	0.489	T3: 716 (> 4.73)	0.78 (0.49–1.23)	1.295
Leucine (g/day)			Leucine (g/day)		
T1:333 (< 6.06)	1.00		T1: 731 (< 5.93)	1.00	
T2: 381 (6.08–7.58)	1.62 (0.98–2.70)	0.060	T2: 683 (5.93–7.46)	0.80 (0.50–1.26)	0.342
T3:353 (> 7.45)	1.47 (0.86–2.51)	0.158	T3:716 (> 7.46)	0.78 (0.49–1.24)	0.306
Isoleucine (g/day)			Isoleucine (g/day)		
T1: 258 (< 3.44)	1.00		T1: 806 (< 2.91)	1.00	
T2: 367 (3.44–4.98)	2.04 (1.14–3.62)	**0.015**	T2: 697 (2.91–4.33)	0.61(0.38–1.25)	0.230
T3: 442 (> 4.98)	1.34 (0.75–2.40)	0.309	T3: 622 (> 4.33)	0.91 (0.59–1.41)	0.689

Values in bold indicate statistically significant results (*p* < 0.05)

Regression models were adjusted for age, skin color, smoking, per capita income, physical activity, binge drinking, saturated fat intake, carbohydrate intake, salt intake and BMI

## Discussion

To the best of our knowledge, this is the first prospective study to investigate the impact of BCAA intake on the incidence of hypertension in Brazilian adults. In our population, in stratified analyses by sex, we observed a direct association between the second tertile of total BCAA and isoleucine intake and risk of hypertension among men. Similarly, in analyses stratified by age group, valine and leucine intake in the second tertile was directly associated with hypertension among adults aged 30–39 years, whereas among younger participants (18–29 years), leucine intake in the second tertile was inversely associated with hypertension risk.

A limited number of studies have evaluated the association between BCAA intake and the incidence of hypertension. Similar to our study, Mirmiran et al. [9] found a direct association between total BCAA and valine intake and the incidence of hypertension in 4315 Iranians. Liu et al. [15] found a direct association between the intake of valine, leucine, and isoleucine and the incidence of hypertension in a sample of 8,491 Chinese individuals. Yu et al. [27] and Salimi et al. [28], in a study of 14,883 Chinese and 4,184 Iranian adults, found a direct association between BCAAs (valine, leucine, and isoleucine) consumption and hypertension after adjusting for variables similar to those used in ours. Pallottini and Fisberg [29] evaluated BCAA intake and its association with cardiometabolic risk factors in 2,691 residents of São Paulo, Brazil. The results showed that adults and older adults classified in the highest quartile of BCAA, leucine, valine, and isoleucine intake had a greater chance of having hypertension. Contrary to our findings, Najafi et al. [30] reported no association between isoleucine and leucine consumption and the incidence of hypertension in the Iranian population, comprising 491 cases and 1,964 controls.

It is noted that associations differed across studies and that three of them found an association between all amino acids (BCCA, isoleucine, leucine, and valine) and hypertension [16, 27, 28]. Therefore, the results remain inconclusive; additionally, the studies conducted to date have included the Chinese and Iranian populations, as well as one study from Brazil. The possible reasons for this difference are numerous and may include study design, dietary patterns, race, gender, and other confounding factors.

The age-stratified results suggest that the metabolic effects of BCAA intake may differ across the life course. Among adults aged 30–39 years, intakes of valine and leucine in the second tertile were associated with higher hypertension incidence, possibly reflecting increased metabolic vulnerability in this age range, including early declines in insulin sensitivity, greater visceral adiposity, and heightened cardiometabolic stress, conditions that may potentiate the adverse vascular effects linked to BCAA metabolism [31].

In contrast, among younger participants (18–29 years), leucine intake in the second tertile was inversely associated with hypertension. This protective association may reflect the greater metabolic flexibility characteristic of early adulthood, including higher amino acid oxidation capacity, lower inflammatory burden, and more efficient endothelial and insulin signaling pathways [32]. In this context, individuals in this age group may benefit from the physiological roles of leucine in muscle metabolism without triggering the metabolic pathways implicated in vascular dysfunction observed at older ages.

The median intake of leucine and isoleucine in the study of Najafi et al. [30] was 3.21 and 1.95 g/day. The mean intake of leucine, valine, isoleucine, and total BCAA in the study of Mirmiran et al. [9] was 91.0, 64.0, 52.0, and 207.0 mg/kg/day. For a standard 70 kg man, this would represent a consumption of 6.37, 4.48, 3.64, and 14.49 g/day, respectively. In the study of Liu et al. [16], the mean intake of leucine, valine, and isoleucine was 4.91, 2.88, and 2.49 g/day for men, respectively, and 3.84, 2.56, and 2.16 g/day for women, respectively. In the study by Yu et al. [28], the cut-off values of new-onset hypertension risk, total BCAAs, isoleucine, leucine, and valine were 15.7 g/day, 4.1 g/day, 6.9 g/day, and 4.6 g/day, respectively. In the study by Salimi et al. [28], the average consumption among hypertensive individuals was 5.26, 3.67, 3.06 and 12.16 for leucine, valine, isoleucine, and total BCAA, respectively. In the study by Pallottini et al. [29], the median total BCAA consumption was 175.01 mg/kg/day or 12.25 g/day. Our results showed the median intakes were 6.69, 4.22, 3.74, and 14.80 g/day for leucine, valine, isoleucine, and total BCAA, respectively.

It is possible to observe that the intake values of leucine, isoleucine, valine, and BCAA in our study were similar to those reported by Mirmiran et al. [9], Yu et al. [27], and Salimi et al. [28], although dietary patterns differed. It is important to highlight that the isoleucine consumption values ​​in the study by Najafi et al. [30] were similar to those found in our research; however, the leucine values ​​were well above (6.69 as opposed to 1.95 g/day).

In stratified analyses by sex, we observed a direct association between the second tertile of total BCAA (13.41–16.42 g) intake and isoleucine intake (3.44–4.98 g) with the incidence of hypertension among men. This pattern may indicate a non-linear relationship, in which moderate elevations in BCAA intake are sufficient to trigger adverse metabolic responses such as mTORC1 activation, insulin resistance, and endothelial dysfunction. In contrast, very high intakes may be accompanied by physiological adaptations or genetic factors that modulate these effects. Consistently, Liu et al. [16] found that when intake levels of isoleucine, leucine, and valine, respectively, exceeded 2.49 g/day, 4.91 g/day, and 2.88 g/day in men and 2.16 g/day, 3.84 g/day, and 2.56 g/day in women, the risk of hypertension increased in a non-linear relationship in the Chinese population.

Leucine and isoleucine are classified as ketogenic amino acids because they act as precursors of acetyl-CoA, an important substrate for lipogenesis [13]. Increased plasma BCAA levels can lead to higher concentrations of metabolic intermediates, such as C3 and C5 acylcarnitines, which are associated with the activation of lipogenic pathways, oxidative stress, and insulin resistance [31]. In addition, the exacerbated activation of the mTORC1 complex and S6K1 kinase by BCAAs prevents signaling mediated by insulin receptor substrates (IRS1 and IRS2), compromising the PI3K/Akt pathway and enhancing the development of insulin resistance [12]. Chronic exposure to BCAAs can also stimulate lipid accumulation, primarily through the metabolite 3-hydroxyisobutyrate (3-HIB), derived from valine, which increases transendothelial transport of fatty acids, contributing to tissue lipotoxicity and dysfunction in insulin action [13]. Additionally, the altered degradation of branched-chain amino acids, triggered by increased plasma concentrations, can induce oxidative stress and disrupt nitric oxide synthesis, contributing to endothelial cell dysfunction [33, 34]. Thus, chronic inflammation and insulin resistance may contribute to the association between BCAA and the elevated risk of cardiovascular diseases, including high blood pressure.

Sex-specific hormonal and metabolic differences may be partially responsible for men’s greater vulnerability to the potential adverse metabolic effects of high BCAA intake. One possible explanation for the sex-specific association observed in our study is the cardioprotective role of estrogen in women [35]. Estrogen enhances endothelial function by stimulating nitric oxide production, exerts anti-inflammatory and antioxidant effects [36], and improves insulin sensitivity via PI3K/Akt pathway modulation [37]. These mechanisms may counterbalance the adverse metabolic and vascular effects of high BCAA intake, such as oxidative stress, endothelial dysfunction, and insulin resistance. Therefore, hormonal differences, particularly the protective effects of estrogen, may contribute to the lower susceptibility to BCAA-related hypertension observed in female participants.

Mirmiran et al. [9] found that the main foods contributing to BCAA intake were dairy products (31.5%), followed by cereals (29%), meat (20.5%), and fish (3.4%). In our study, the main foods were unprocessed chicken (16.56%), unprocessed beef (14.98%), dairy products (16.33%), fish (7.85%), and beans/lentils (6.44%). In the study by Pallotini et al. [29] with a Brazilian dietary pattern, the main sources of BCAA were also unprocessed beef (20.3%) and unprocessed poultry (14.2%). In the study by Yu et al. [27], the top five main food sources of the three types of BCAAs were cereals, red meat, beans, fish and seafood, and vegetables; however, the percentages were not reported. Liu et al. [15] did not analyze the main foods contributing to BCAA intake. The results showed that differences in BCCA intake correspond to distinct dietary patterns. It should be noted that all six studies assessed food consumption by using a food frequency questionnaire.

A Brazilian study based on a nationally representative sample identified three main dietary patterns. The first was characterized by high consumption of fruits and vegetables; the second was a traditional pattern encompassing beans, red meat, fish, and poultry; and the third was a Western diet, marked by intake of soft drinks, sweets, sandwiches, snacks, or pizza [38]. These findings indicate that our population follows a mixed diet profile. One possible explanation for the absence of an association between BCAA consumption and hypertension in women is the protective effect of certain dietary componentes such as fruit, vegetables, and low-fat dairy products—foods commonly consumed in the Minas Gerais region and likely present in the CUME cohort. This hypothesis is supported by our analysis showing that greater adherence to the Healthy Plant-Based Diet Index (hPDI) was associated with a 45% reduction in the risk of hypertension [39]. Therefore, the higher hPDI scores and overall better diet quality observed among women in the CUME study may help explain the lack of association between BCAA intake and hypertension in this group (Online Resource 3).

It is important to note that the contradictory findings across studies may be attributed to methodological differences, population variations, dietary sources, and the complexity of amino acid interactions. However, we made adjustments for considerable variables from the literature.

The present study is the first to evaluate the impact of BCAA on the incidence of hypertension in Brazilians. The strengths include its cohort design, sample size, and model adjustment for multiple variables. One limitation was the lack of plasma BCAA measurements, which precluded further evaluation of the influence of BCAA intake on body concentrations. Hypertension at follow-up was defined based on a single report of elevated blood pressure, use of antihypertensive medication, or physician diagnosis. Although repeated measurements are recommended in clinical settings to confirm hypertension, this approach is not always feasible in extensive epidemiological studies. This classification strategy may have introduced some degree of misclassification. However, the high agreement observed in validating self-reported hypertension in our cohort supports the reliability of this measure.

The 144-item food frequency questionnaire (FFQ) used in this study has been previously validated to estimate overall dietary intake in our population; however, it was not specifically designed or validated to assess individual amino acid intake, including BCAAs. This limitation is inherent to most FFQs, given the complexity involved in accurately quantifying specific nutrient subtypes. Furthermore, we did not perform repeated dietary assessments during the follow-up period, which limited our ability to capture potential changes in dietary habits over time. Consequently, our analyses are based on baseline dietary data, assuming relative stability in dietary patterns over the study period. This approach may result in some misclassification of exposure, potentially attenuating the observed associations. Finally, the possibility of residual confounding arises from unmeasured lifestyle factors.

## Conclusion

In conclusion, this prospective analysis provides novel evidence that moderate BCAA intake, particularly total BCAAs and isoleucine among men, as well as valine and leucine among adults aged 30–39 years, may contribute to the development of hypertension in Brazilian adults. Although the sex-specific association observed for isoleucine warrants cautious interpretation, plausible biological mechanisms may be involved, including sex differences in muscle mass, hormonal modulation of amino acid metabolism, and distinct dietary patterns associated with BCAA intake in men. Experimental and clinical research also suggests that BCAAs may influence insulin resistance, endothelial function, and activation of metabolic pathways such as mTOR, potentially contributing to elevations in blood pressure.

From a public health perspective, these findings highlight the importance of monitoring high-protein dietary patterns and the growing use of BCAA-containing supplements, especially among young adults and men. Incorporating guidance on balanced protein sources within dietary recommendations may contribute to hypertension prevention strategies. Further research is needed to clarify the mechanistic pathways involved, evaluate potential threshold effects, and determine whether modifications in BCAA consumption could be integrated into clinical and population-based interventions aimed at reducing hypertension risk.

## Supplementary Information

Below is the link to the electronic supplementary material.

Supplementary Material 1
