# Supplementary material for: Hypertension incidence according to branched-chain amino acid intake in Brazilian adults: a six-year follow-up of the CUME study
Source: Eur J Nutr. 2026 Mar 11;65(3):91. doi: 10.1007/s00394-026-03903-1 (PMC12979256; doi:10.1007/s00394-026-03903-1)
Supplement: Supplementary file 1 — Supplementary Material 1 [file 394_2026_3903_MOESM1_ESM.docx]

**Online Resource**

**Hypertension incidence according to branched-chain amino acid intake in Brazilian adults: A six-year follow-up of the CUME Study**

Fernanda Maria Oliveira da Silva, PhD^ab^, Adriano Marçal Pimenta, PhD ^c^, Leidjaira Lopes Juvanhol, PhD.^d^, Helen Hermana Miranda Hermsdorff, PhD ^ab^, and Josefina Bressan, PhD ^ab^

aLaboratory of Energy Metabolism and Body Composition, Department of Nutrition and Health, Federal University of Viçosa, Viçosa. MG, Brazil.

bLaboratory of Clinical Analysis and Genomics, Department of Nutrition and Health, Federal University of Viçosa, Viçosa. MG, Brazil.

cDepartment of Nursing, Federal University of Paraná, Curitiba, Brazil.

dDepartment of Nutrition and Health, Federal University of Viçosa, Viçosa, Brazil.

Corresponding author: Josefina Bressan, Department of Nutrition and Health, Federal University of Viçosa, Viçosa, Minas Gerais, Brazil. E-mail: [jbrm@ufv.br](mailto:jbrm@ufv.br)


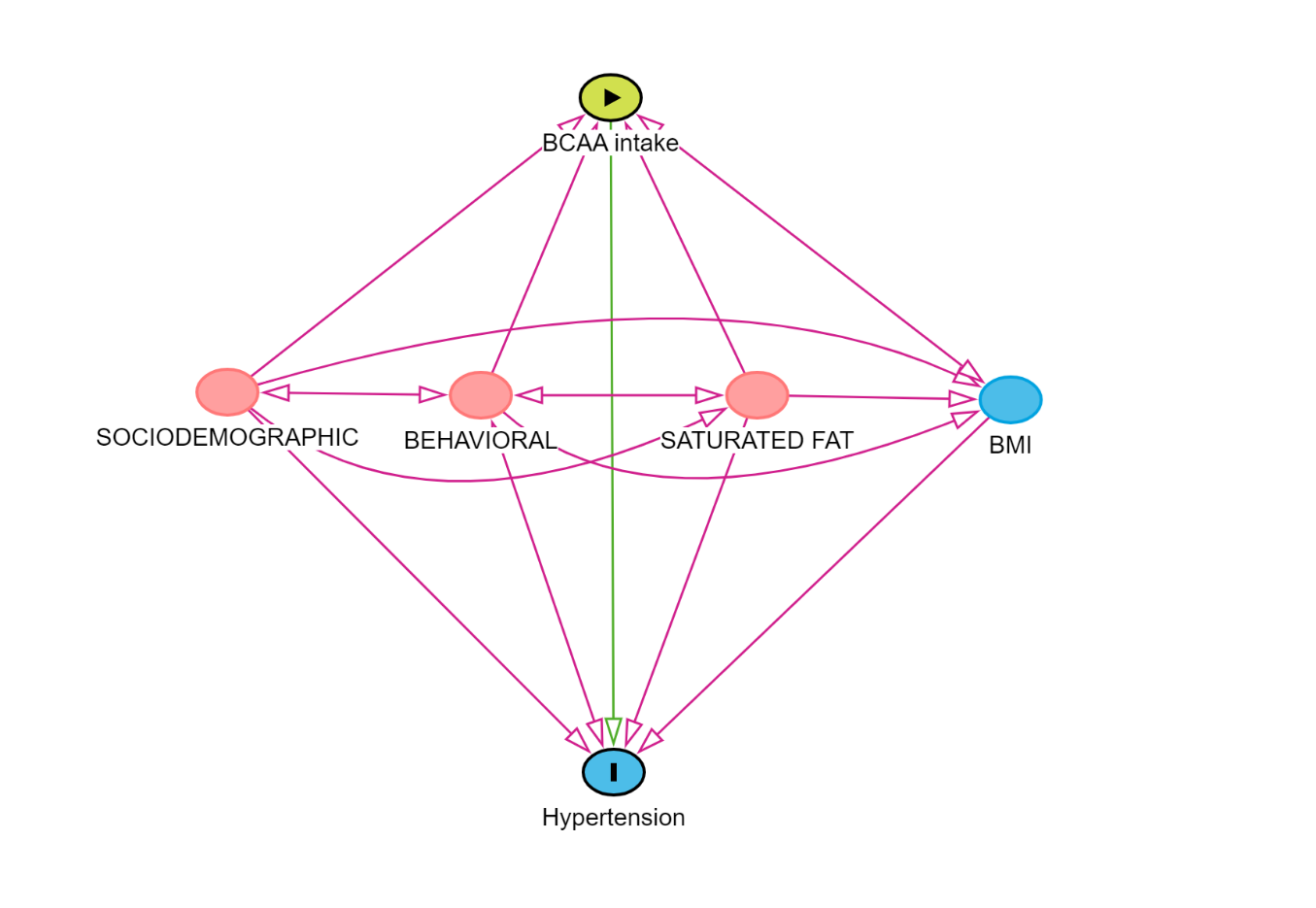


**Online resource 1.** Acyclic graph depicting the association between branched-chain amino acid (BCAA) intake and obesity. Hypertension is the outcome variable. BCAA (total, valine, leucine, and isoleucine) intake is the exposure variable. Sociodemographic (sex, age, skin color, and per capita income), behavioral (physical activity, alcohol intake, smoking), and BMI (body mass index) characteristics are the covariates. Green arrows indicate causality, pink arrows indicate ancestors of the outcome and exposure variables, and potential adjustment variables.

| Online Resource 2 – Association between branched-chain amino acid intake tertiles and hypertension incidence (CUME study, n = 3192, 2016-2022). | | | | | | |
| --- | --- | --- | --- | --- | --- | --- |
| **Variables** | **BCAA intake tertiles** | | | | | **Trend p-value** |
|  | **T1**  **n = 1064** | **T2**  **n = 1064** | | | **T3**  **n = 1064** |  |
|  | **HR** | **HR (95% CI)** | | | **HR (95% CI)** |  |
| **Total BCAA (g/day)** | **<13.24** | **13.24-16.41** | | | **>16.41** |  |
| Crude | 1.00 | 1.14 (0.83-1.58) | | | 0.95 (0.68-1.33) | 0.788 |
| Adjusted | 1.00 | 0.78 (0.47-1.30) | | | 0.63 (0.37-1.09) | 0.100 |
|  | **HR** | **HR (95% CI)** | | | **HR (95% CI)** |  |
| **Valine (g/day)** | **<3.76** | | **3.76-4.72** | **>4.72** | |  |
| Crude | 1.00 | 1.07 (0.78-1.48) | | | 0.92 (0.66-1.29) | 0.155 |
| Adjusted | 1.00 | 0.78 (0.47-1.29) | | | 0.63 (0.36-1.09) | 0.099 |
|  | **HR** | **HR (95% CI)** | | | **HR (95% CI)** |  |
| **Leucine (g/day)** | **<5.98** | **5.98-7.46** | | | **>7.46** |  |
| Crude | 1.00 | 1.09 (0.79-1.51) | | | 1.00 (0.71-1.40) | 0.983 |
| Adjusted | 1.00 | 0.66 (0.40-1.11) | | | 0.69 (0.41-1.17) | 0.149 |
|  | **HR** | **HR (95% CI)** | | | **HR (95% CI)** |  |
| **Isoleucine (g/day)** | **<3.08** | | **3.08-4.57** | **>4.57** | |  |
| Crude | 1.00 | 1.08 (0.77-1.51) | | | 1.21 (0.87-1.69) | 0.235 |
| Adjusted | 1.00 | 0.52 (0.55-1.54) | | | 0.82 (0.48-1.39) | 0.465 |

The regression model was adjusted for age, skin color, smoking, per capita income, physical activity, excessive alcohol consumption, saturated fat intake, carbohydrate intake, salt intake, and additionally, COVID-19.

HR, Hazard Ratio.

95% CI, 95% Confidence Interval.

BCAA, branched-chain amino acids.

| **Online Resource 3** - Sex of the participants according to the health plant-based index (hPDI) quintiles (CUME Study, *n* =3192, 2016–2022) | | | | | | |
| --- | --- | --- | --- | --- | --- | --- |
| Health plant-based diet index (hPDI) | | | | | | |
|  | Quintile 1 | Quintile 2 | Quintile 3 | Quintile 4 | Quintile 5 | P-value |
| **Variable** |  |  |  |  |  |  |
| **Sex** |  |  |  |  |  | **< 0.001** |
| Male | 308 (28.87) | 230 (21.56) | 210 (19.68) | 197 (18.46) | 122 (11.43) |  |
| Female | 440 (20.71) | 381 (17.93) | 428 (20.14) | 459 (21.60) | 417 (19.62) |  |

| **Online Resource 4** – Association between branched-chain amino acid (BCAA) intake tertiles and hypertension incidence, stratified by age (CUME Study, n = 3192, 2016–2022). | | | | | | | | | | | | | | |
| --- | --- | --- | --- | --- | --- | --- | --- | --- | --- | --- | --- | --- | --- | --- |
|  | **18-29**  **(n=940)** |  |  | **30-39**  **(n=1322)** |  |  | **40-49**  **(n=603)** |  |  | **50-59**  **(n= 261)** |  |  | **>60**  **(n=66)** |  |
|  | **HR (95% CI)** | **p-Value** |  | **HR (95% CI)** | **p-Value** |  | **HR (95% CI)** | **p-Value** |  | **HR (95% CI)** | **p-Value** |  | **HR (95% CI)** | **p-Value** |
| **BCAA (n) (g/day)** |  |  | **BCAA (n) (g/day)** |  |  | **BCAA (n) (g/day)** |  |  | **BCAA**  **(n) (g/day)** |  |  | **BCAA (n) (g/day)** |  |  |
| T1:299 (<13.39) | 1.00 |  | T1:417 (<13.40g) | 1.00 |  | T1:220 (<13.04g) | 1.00 |  | T1:104  (<12.37g) | 1.00 |  | T1:24 (<12.81g) | 1,00 |  |
| T2:307  (13.39-16.63) | 0.49 (0.20-1.16) | 0.108 | T2:467  (13.40-16.38) | 1.77 (0.98-3.00) | 0.056 | T2:197  (13.04-16.24) | 1.01 (0.57-2.11) | 0.771 | T2:74  (12.37-16.13) | 1.99 (0.76-5.20) | 0.159 | T2:19  (12.81-17.00) | 2.28 (0.25-20.21) | 0.458 |
| T3:334 (>16.63) | 0.82 (0.37-1.84) | 0.648 | T3:438 (>16,38) | 1.05 (0.54-2.04) | 0.865 | T3:186  (>16.24) | 1.10 (0.58-2.10) | 0.756 | T3:83 (>16.13) | 0,55 (0,16-1.84) | 0.336 | T3:23  (>17.00) | 1.95 (0.20-18.20) | 0.556 |
| **Valine**  **(n) (g/day)** |  |  | **Valine**  **(n) (g/day)** |  |  | **Valine**  **(n) (g/day)** |  |  | **Valine (n) (g/day)** |  |  | **Valine (n) (g/day)** |  |  |
| T1:299 (<3.82) | 1.00 |  | T1: 420  (<3.81) | 1.00 |  | T1:215  (<3.70) | 1.00 |  | T1:107  (<3.51) | 1.00 |  | T1:23 (<3.65) | 1.00 |  |
| T2:305  (3.82-4.82) | 0.47 (0.20-1.12) | 0.089 | T2: 466  (3.81-4.71) | 1.74 (1.01-3.02) | **0.045** | T2: 200  (3.70-4.64) | 1.07 (0.54-2.11) | 0.829 | T2:83  (3.51-4.68) | 1.23 (0.45-3.37) | 0.674 | T2:20  (3.65 -4.90) | 2.19 (0.25-19.04) | 0.475 |
| T3: 336  (>4.82) | 0.74 (0.33-1.67) | 0.474 | T3:436 (>4.71) | 0.99 (0.51-1.92) | 0.989 | T3:188  (>4.64) | 1.27 (0.66-2.45) | 0.465 | T3:81  (>4.68) | 0.46 (0.14-1.47) | 0.191 | T3:23 (>4.90) | 1.89 (0.20-17.12) | 0.570 |
| **Leucine (n) (g/day)** |  |  | **Leucine**  **(n) (g/day)** |  |  | **Leucine (n) (g/day)** |  |  | **Leucine (n) (g/day)** |  |  | **Leucine**  **(n) (g/day)** |  |  |
| T1:292  (<6.08) | 1.00 |  | T1:419 (<6.05) | 1,00 |  | T1: 222 (<5.80) | 1.00 |  | T1: 108(<5.51) | 1.00 |  | T1:23  (<5.57) | 1.00 |  |
| T2:312  (6.08-7.59) | 0.36 (0.14-0.89) | **0.028** | T2: 467  (6.05-7.45) | 1.94 (1.10-3.41) | **0.021** | T2: 188 (5.80-7.34) | 0.78 (0.39-1.58) | 0.505 | T2: 77  (5.51-7.29) | 2.31 (0.87-6.09) | 0.091 | T2:20  (5.57-7.65) | 2.19 (0.25-19.04) | 0.475 |
| T3:336  (>7.59) | 0.73 (0.33-1.60) | 0.436 | T3: 436 (>7.45) | 1.08 (0.55-2.13) | 0.805 | T3:193 (>7.34) | 1.30 (0.69-2.42) | 0.405 | T3: 76 (>7.29) | 0.71 (0.21-2.40) | 0.590 | T3: 23 (>7.65) | 1.89 (0.20-17.12) | 0.570 |
| **Isoleucine**  **(n) (g/day)** |  |  | **Isoleucine**  **(n) (g/day)** |  |  | **Isoleucine**  **(n) (g/day)** |  |  | **Isoleucine**  **(n) (g/day)** |  |  | **Isoleucine**  **(n) (g/day)** |  |  |
| T1:301  (<3.14) | 1.00 |  | T1: 456 (<3.03) | 1.00 |  | T1:200  (<3.08) | 1.00 |  | T1: 90  (<2.98) | 1,00 |  | T1: 17 (<3.25) | 1.00 |  |
| T2: 334  (3.14-4.54) | 0.75 (0.32-1.72) | 0.498 | T2: 433  (3.03-4.54) | 1.05 (0.57-1.90) | 0.871 | T2: 192  (3.08-4.69) | 0.76 (0.40-1.45) | 0.408 | T2: 80  (2.98-4.69) | 2.04 (0.70-5.96) | 0.188 | T2: 25  (3.25-4.83) | __^a^ |  |
| T3: 335  (>4.54) | 0.66 (0.28-1.53) | 0.336 | T3: 433 (>4.54) | 1.16 (0.65-2.05) | 0.603 | T3: 211  (>4.69) | 0.59 (0.31-1.12) | 0.110 | T3: 91 (>4.69) | 1.44 (0.48-4.30) | 0.505 | T3: 24 (>4.83) | __^a^ |  |

Notes: a: non-convergent model.
HR, Hazard Ratio.

95% CI, 95% Confidence Interval
